# Does the course of Val122Ile differ from SSA, or is selection bias a factor?

**DOI:** 10.1186/1750-1172-10-S1-P18

**Published:** 2015-11-02

**Authors:** Hallie Geller, Tara Mirto, Rodney Falk

**Affiliations:** 1 Brigham and Women's Hospital, Cardiac Amyloidosis Program, 02115, Boston, MA, USA

## Hypothesis

There are conflicting data regarding survival in Val122Ile versus SSA cardiac amyloidosis, suggesting either better survival in SSA or similar survival between the groups. Since survival is often calculated from time of diagnosis, a delayed diagnosis in Val122Ile from symptom onset would bias survival toward SSA. We therefore sought to determine whether patients with symptomatic SSA and Val122Ile seen at our Program showed different patterns of referral, and whether one group had a longer time to diagnosis than the other.

## Methods

Records of all 30 Val122Ile patients with cardiac involvement seen over an 8 year period were compared to records of 34 randomly selected patients with cardiac SSA. We estimated onset of cardiac symptoms likely due to amyloidosis from detailed history review and determined definitive diagnosis based on biopsy or (for Val122Ile) either biopsy or typical echocardiogram with positive genetic testing. Referral patterns were also determined, specifically whether patients were from within our home state or referred from out-of-state. Death was determined by national database of deaths, medical records or on-line obituary search if patient had been lost to follow-up.

## Results

Mean age at diagnosis did not differ between groups: Val122Ile =71.4 yr, SSA =73.9 yr.

There was a wide range of symptom duration between symptom onset and diagnosis, ranging from weeks to 9 years in both groups, with a slightly longer duration of symptoms in the SSA group than in Val122Ile (32.4 v 21.6 months p=ns). Actuarial survival from diagnosis did not differ between groups, being 33 months in Val122Ile and 36 months in SSA, but there was a a strong trend to better survival from symptom onset in SSA (66 months in SSA v 51 months in Val122Ile). Referral pattern differed significantly between the 2 groups, with 63% of Val122Ile patients referred from in-state compared to only 34% of SSA (p<0.05).

## Conclusion

The higher rate of out-of-state referrals for SSA may suggest either an increased awareness of the disease locally, or possibly a socioeconomic bias in favor of SSA patients. The trend toward earlier diagnosis from symptom onset in Val122Ile patients may affect analysis of survival comparison with SSA if date of diagnosis is used in calculating survival, but the overall median survival in both groups, whether from diagnosis or symptom onset remains poor, and underscores the pressing need for disease-modifying drug trials in both groups.

